# Effects of Different Photoperiods on Peripheral 5-Hydroxytryptamine Metabolism, Breast Muscle Glucose Metabolism, and Myopathies in Broilers

**DOI:** 10.3390/metabo14100567

**Published:** 2024-10-21

**Authors:** Miao Yu, Mengjie Xu, Guangju Wang, Jinghai Feng, Minhong Zhang

**Affiliations:** 1 State Key Laboratory of Animal Nutrition and Feeding, Institute of Animal Sciences, Chinese Academy of Agricultural Sciences, Haidian, Beijing 100193, China; 82101211223@caas.cn (M.Y.); 82101215394@caas.cn (M.X.); guangju.wang@wur.nl (G.W.); fengjinghai@caas.cn (J.F.); 2 Adaptation Physiology Group, Wageningen University and Research, 6708 PB Wageningen, The Netherlands

**Keywords:** long photoperiod, peripheral 5-hydroxytryptamine metabolism, glucose metabolism, glycolysis, white striping, wooden breast, broiler

## Abstract

**Background**: There is a close relationship between breast muscle glucose metabolism, peripheral 5-hydroxytryptamine (5-HT), and myopathies in animals. Here, this study aimed to investigate the effects of different photoperiods on peripheral 5-HT metabolism, white striping (WS), and wooden breast (WB) in broilers. **Methods**: A total of 216 healthy 5-day-old (d) Arbor Acres (AA) male broilers were randomly assigned to 12L:12D, 18L:6D, and 24L:0D photoperiods for 4 weeks. **Results**: Compared with the 12L:12D photoperiod, we found the WB score in broilers was significantly increased in the 18L:6D and 24L:0D photoperiod at week 4 (*p* < 0.05). Muscle glycogen was significantly reduced (*p* < 0.05) and glycolysis was promoted in the breast muscles of broilers under the 18L:6D and 24L:0D photoperiods at week 2 and 4. Peripheral 5-HT concentrations, the mRNA expression of tryptophan hydroxylase 1 (TPH1) and serotonin transporter (SERT) in the cecal mucosa, and 5-hydroxytryptamine receptor 2A (5-HTR_2A_) mRNA expression in the breast muscle of broilers significantly up-regulated in the 18L:6D and 24L:0D photoperiod at week 2 and 4 (*p* < 0.05). **Conclusions**: Our findings revealed that extending the photoperiod improved the breast muscle growth rate, but up-regulated 5-HT synthesis and secretion to higher peripheral 5-HT, induced breast muscle glucose metabolism disorder, and increased WB incidence rates in broilers.

## 1. Introduction

In recent years, the incidence rate of white striping (WS) and wooden breast (WB) myopathies has increased yearly in broilers, which cause meat quality deterioration and production efficiency reduction, seriously impairing the nutritional contents of chicken and the economic benefits of the broiler industry [1]. Exposure to long photoperiods acts as an important environmental factor in promoting growth rates [2,3,4]. The rapid growth rate of breast muscle leads to insufficient oxygen supply in meeting muscle growth, inducing hypoxic stress and abnormal energy metabolism, which is the main reason for the occurrence of WS and WB myopathies in broilers [5,6,7,8,9]. It has been reported that the occurrence of WS and severe WS breasts were lower in exposure to a 14L:10D photoperiod than an 18L:6D photoperiod [10].

5-HT is an important neurotransmitter and vital precursor for the hormone melatonin in birds and mammals [11,12,13]. The most studied and best understood is the 5-HT system in the central nervous system in broilers [14,15]. Peripheral 5-HT is a monoamine neurotransmitter involved in various physiological activities in peripheral tissues, including meat quality regulation of skeletal muscle. Previous research has observed that peripheral 5-HT was closely related to breast meat quality in broilers, with negative correlations between cooking loss, redness, and 5-HT levels, and positive correlations between muscle pH, off-flavor intensity, and 5-HT levels could be found in the breast muscle of broilers [16]. Exposure to long photoperiods in broilers could cause oxidative stress [17,18], and more is known about stress increasing the 5-HT concentrations in the intestinal tract [19,20]. Nearly 90% of peripheral 5-HT is produced by enterochromaffin cells in the intestinal mucosa [21,22]. However, so far, there are currently no studies on the effects of different photoperiods on peripheral 5-HT metabolism of broilers.

Moreover, glucose metabolism is also deeply linked with WS and WB myopathies and peripheral 5-HT. There were previous studies that suggested the occurrence of WS and WB myopathies in broilers accompanied by glycogen synthesis inhibition and glycolytic activity reduction [8,23,24]. Moreover, Peripheral 5-HT has been also observed to be closely related to glucose intake and glycolysis in skeletal muscle cells of rats through activating 5-HTR_2A_ in vitro [25,26,27]. Based on the research reviewed above, we proposed a hypothesis that peripheral 5-HT metabolism, glucose metabolism, and myopathies of breast muscle in broilers are closely related under different photoperiods.

The breast muscle is a crucial part of the edible portion of broilers, serving as the most economically valuable tissue. In this study, we aimed to examine the effects of different photoperiods on peripheral 5-HT metabolism, glucose metabolism of breast muscle, and WS and WB myopathies in broilers, so as to provide new insights and potential targets for improving the growth and meat quality of breast muscles in broilers.

## 2. Materials and Methods

This research received approval from the Institutional Ethics Committee of Experiment Animal Welfare and Ethics at the Institute of Animal Science of the Chinese Academy of Agricultural Sciences (CAAS) (permit number: IAS 2022-117).

### 2.1. Birds and Housing

A total of 1 d 300 AA male broilers were purchased from a commercial hatchery (Luanping Yijia Agricultural Development Co., Ltd., Chengde, China). The broilers were raised until 5 days old according to the standard of the *Arbor Aces Broiler Management Handbook* [28]. A total of 5 d 216 AA male broilers with similar body weight (75 g ± 10) were randomly assigned to 3 photoperiod treatment groups with 6 replicates per treatment, with 12 broilers per replicate. The 3 groups received different photoperiods with 12L:12D (12 h light and 12 h dark), 18L:6D (18 h light and 6 h dark), and 24L:0D (24 h light) for 4 weeks, respectively. The light intensity was 15 lux. The light condition of broilers was mainly controlled by environmental factor perception equipment and environmental factor control equipment. All broilers were housed in stainless single-layer flat cages without roofs (0.82 m width × 0.70 m length × 0.60 m height).

The experimental period lasted for 4 weeks (5–33 d). As per the Zeitgeber Time system, 8:00 am at d 1 of this experiment is recorded as ZT0. The light period of the 12L:12D photoperiod spans from ZT0 to ZT12 and the dark period spans from ZT13 to ZT24. The light period of the 18L:6D photoperiod spans from ZT0 to ZT18 and the dark period spans from ZT19 to ZT24. The light period of the 24L:0D photoperiod spans from ZT0 to ZT24. The environmental temperature and humidity were carried out according to the standard of the *Arbor Aces Broiler Management Handbook* [28]. The relative humidity was maintained at 60%.

**Figure 1:**
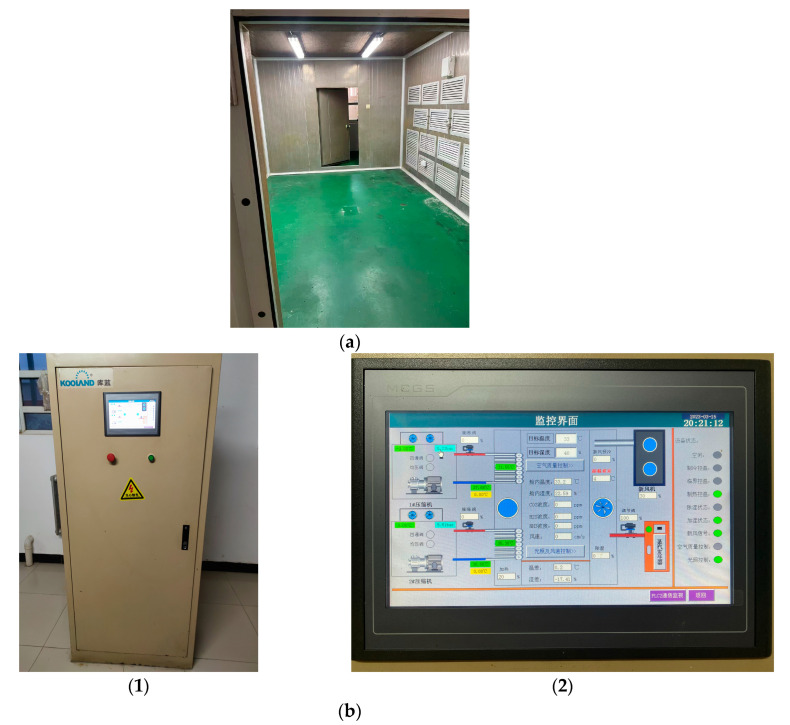
The artificial climate chamber and the environmental factor perception control equipment. (**a**) Artificial climate chamber. (**b**) Environmental factor perception control equipment: (**1**) the equipment and (**2**) the perception-control panel.

All broilers were raised in the artificial climate chambers (4.08 m × 2.88 m × 2.38 m) of the State Key Laboratory of Animal Nutrition and Feeding, Chinese Academy of Agricultural Sciences (Figure 1). Except for the photoperiods in the artificial climate chambers, other environmental parameters remained the same.

### 2.2. Diets and Feeding Program

All broilers received a standard corn–soybean meal basal diet in three feeding programs for 4 weeks (5–7 d, 8–21 d, and 22–33 d) (Table 1) [29], formulated according to AA broiler recommendations [30]. All broilers had free access to experimental diets and water.

### 2.3. Sample Collection

At the end of week 2 and week 4 of the trial period, one broiler from each replicate was selected with a body weight close to the average after 12 h of feed deprivation. All samples of broilers were collected at ZT1. Blood samples were obtained via vacuum blood collection tubes from the wing vein of broilers. The serum was separated by blood using centrifugation at 3000 r/min for 10 min at 4 °C, and the supernatant was collected and stored at −20 °C until it was used for biochemical analysis. Then, the broilers from each group were killed by carbon dioxide (CO_2_). Tissue samples of left breast muscle and the cecal mucosa rinsed with sterile normal saline (NaCl 9 g/L) were immediately collected and snap-frozen in liquid nitrogen, then kept in a −80 °C freezer for measurements of gene expression analysis and biochemical analysis.

### 2.4. Growth Performance

At the end of week 2 and week 4 in the trial period, the provided and residual feed amount and broiler body weight of each replicate were recorded. The average body weight and average body weight gain (average body weight gain = final body weight − initial body weight) were calculated. The right breast muscle of one broiler chicken from each replicate was randomly selected to determine the breast muscle weight/breast muscle ratio (breast muscle ratio = breast muscle weight/body weight × 100%), breast muscle weight gain (breast muscle weight gain = final breast muscle weight − initial breast muscle weight), and breast muscle weight gain ratio (breast muscle weight gain ratio = breast muscle weight gain/body weight gain × 100%).

### 2.5. White Striping and Wooden Breast Score

White striping scoring method: normal breast muscle scored 0 points (no obvious white stripes); mild white striping scored 1 point (few and less than 1 mm thick white stripes); severe white striping scored 2 points (lot and more than 1 mm thick white stripes).

Wooden breast scoring method: normal breast muscle scored 0 points (the breast muscle is soft with a normal appearance, and both ends of the breast muscle can hang freely when placed in the hand); mild wooden breast scored 1 point (the hard part of breast muscle is mainly concentrated in the top area); moderate wooden breast scored 2 points (the top half part of breast muscle is hardened, the middle-to-bottom part of the breast muscle is still a certain softness); severe wooden breast scored 3 points (the breast muscle is hard to the touch without softness, the surface presents with woody texture).

### 2.6. Peripheral 5-HT Concentrations Analysis of the Serum, Cecal Mucosa, and Breast Muscle

The 5-HT concentrations in serum, cecal mucosa, and breast muscle were measured using ELISA kits (KJ00367CH, Kangjia Hongyuan Biotechnology Co., Ltd., Beijing, China) by the Multiskan MK3 microplate reader (Thermo Fisher Scientific, Waltham, MA, USA) following the instructions of the manufacturer. The cecal mucosa and breast muscle were transferred into a glass homogenizer and 5–10 mL of pre-cooled PBS buffer (1:5 mass/volume ratio of tissue to PBS buffer is recommended) was added for thorough grinding, followed by centrifugation of the prepared homogenate at 3500 r/min for 15 min. The resulting supernatant was retained for assay. This kit was specifically used to detect 5-HT and showed no obvious cross-reactivity with other similar substances. This kit was also suitable for the pan-species (general). Three parallels were made for each sample.

### 2.7. Glucose Metabolism Analysis of the Breast Muscle

The glycogen concentrations in breast muscle were determined using a glycogen assay kit (TY-1-Y, Suzhou Keming Biotechnology Co., Ltd., Suzhou, China) based on the anthrone method following the instructions of the manufacturer by anthrone assay. The breast muscle samples (0.1 g) were boiled in 375 μL of 30% KOH for 20 min. The optical density was read at 620 nm with the Multiskan MK3 microplate reader (Thermo Fisher Scientific, Waltham, MA, USA).

The lactic acid concentrations in breast muscle were determined using the Lactic Acid assay kit (A019-2-1, Nanjing Jiancheng Bioengineering Institute, Nanjing, China) following the instructions of the manufacturer. The optical density was read at 530 nm with the Multiskan MK3 microplate reader (Thermo Fisher Scientific, Waltham, MA, USA). The recovery rate of pyruvate could reach 100%.

The pyruvate concentrations in breast muscle were tested using a pyruvate assay kit (A081, Nanjing Jiancheng Bioengineering Institute, Nanjing, China) following the instructions of the manufacturer. The optical density was read at 505 nm with the Multiskan MK3 microplate reader (Thermo Fisher Scientific, Waltham, MA, USA). The recovery rate of pyruvate could reach 96%.

We transferred the breast muscle into a glass homogenizer and added 5–10 mL of pre-cooled PBS buffer (1:5 mass/volume ratio of tissue to PBS buffer is recommended) for thorough grinding, centrifuged the prepared homogenate at 3500 r/min for 15 min, and then retained the supernatant for assay. All kits were specifically used to detect lactic acid and showed no obvious cross-reactivity with other similar substances. These kits were suitable for the pan-species (general) in all tissues.

We then proceeded to determine the L/P (L/P = lactic acid concentrations /pyruvate concentrations).

### 2.8. Total RNA Extraction and Reverse Transcription Analysis and Quantitative Real-Time PCR

The mRNA expression of TPH1, SERT, and MAOA in cecal mucosa, and 5-HTR_2A_ in breast muscle was analyzed by quantitative real-time PCR. Total RNA was isolated from the cecum, cecal mucosa, and breast muscle employing Trizol Reagent, followed by reverse transcription utilizing the Prime Script RT Reagent Kit with gDNA Eraser from Takara (Dalian, China). Quantitative real-time PCR was conducted using TB Green Premix Ex Taq II (Takara) on a Light Cycler 96 PCR System to assess mRNA levels. Gene expression was measured through relative RT-PCR, with the primer sequences detailed in Table 2. The primers used are shown in Table 2. Relative quantification of gene expression was determined by 2^−ΔΔCt^ and Recombinant Glyceraldehyde-3-Phosphate Dehydrogenase (GAPDH) acted as the control gene.

### 2.9. Statistical Analysis

The data from the experiment were analyzed using a one-way ANOVA, performed using SPSS 23.0 (SPSS Inc., Chicago, IL, USA). The figures of this study were generated by using GraphPad Prism 8.0 (GraphPad Inc., San Diego, CA, USA). Replicate (*n* = 6) served as the experimental unit. The results in the tables are shown as the mean. *p* > 0.05 and *p* < 0.05 were deemed the statistical non-significance and significance thresholds, respectively.

## 3. Results

### 3.1. Effects of Different Photoperiods on Growth Performance in Broilers

To explore the effects of different photoperiods on the growth performance of the breast muscle in broilers, we analyzed the growth rate of breast muscle. As shown in Table 3, the higher average body weight and breast muscle weight were observed underlying extended light photoperiods. Compared with the 12L:12D photoperiod, the average body weight and breast muscle weight of broilers significantly increased in the 18L:6D photoperiod and the 24L:0D photoperiod during week 0 to week 2 and week 0 to week 4 (*p* < 0.05). The breast muscle ratio of broilers was also significantly enhanced in the 18L:6D photoperiod and the 24L:0D photoperiod during week 0 to week 2 in comparison with the 12L:12D photoperiod (*p* < 0.05). Compared with the 12L:12D photoperiod, the breast muscle ratio of broilers in the 24L:0D photoperiod from week 0 to week 4 was significantly improved (*p* < 0.05), but there was no effect on the breast muscle ratio of broilers in the 18L:6D photoperiod (*p* > 0.05). As also depicted in Table 3, compared with the 12L:12D photoperiod, the average body weight gain and breast muscle weight gain of broilers were markedly increased in the 18L:6D photoperiod and the 24L:0D photoperiod from week 2 to week 4 (*p* < 0.05). However, there was no effect on the breast muscle weight gain ratio of broilers in the three photoperiods from week 2 to week 4 (*p* > 0.05). Furthermore, the feed intake per broiler was also markedly increased in the 18L:6D photoperiod and the 24L:0D photoperiod in the whole experiment (*p* < 0.05).

### 3.2. Effects of Different Photoperiods on White Striping and Wooden Breast Score in the Breast Muscle of Broilers

To explore the effects of different photoperiods on the myopathies of breast muscle in broilers, we evaluated the white striping and wooden breast score. As depicted in Table 4, compared with the 12L:12D photoperiod, the white striping score of broilers had no marked effects in the 18L:6D photoperiod and the 24L:0D photoperiod at week 2 and week 4 (*p* > 0.05), respectively. Compared with the 12L:12D photoperiod, the wooden breast score of broilers was significantly raised in the 18L:6D photoperiod and the 24L:0D photoperiod at week 4 (*p* < 0.05), but there were no effects on those at week 2 (*p* > 0.05).

### 3.3. Effects of Different Photoperiods on Glucose Metabolism in the Breast Muscle of Broilers

To investigate the impact of different photoperiods on glucose metabolism status, we measured the products of glucose metabolism in the breast muscle. As depicted in Table 5, compared with the 12L:12D photoperiod, following treatment with extending light exposure, the muscle glycogen of broilers significantly reduced in the 18L:6D photoperiod and the 24L:0D photoperiod at week 2 and week 4 (*p* < 0.05). Compared with the 12L:12D photoperiod, lactic acid and pyruvate concentrations and the L/*p* in the breast muscle of broilers increased significantly in the 18L:6D photoperiod and the 24L:0D photoperiod at week 2 and week 4 (*p* < 0.05).

### 3.4. Effects of Different Photoperiods on Peripheral 5-HT Metabolism of Broilers

**Figure 2:**
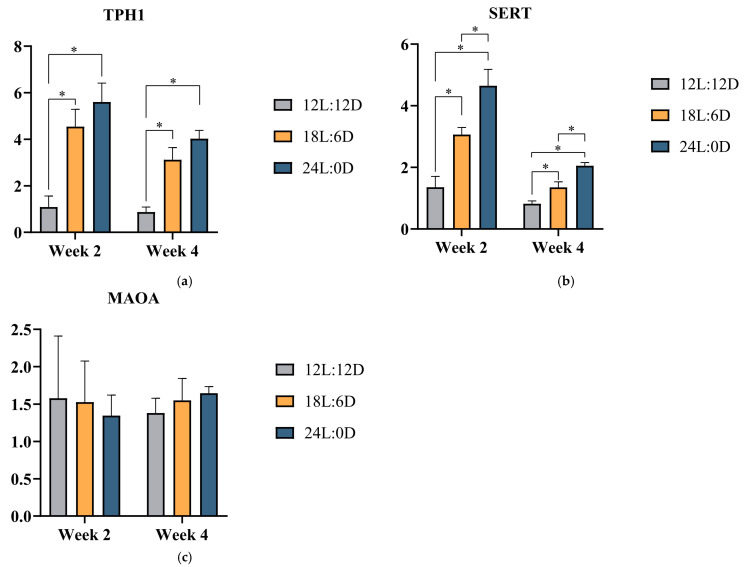
Effects of different photoperiods on 5-HT metabolism-related genes mRNA expression levels in the cecal mucosa of broilers. (**a**) TPH1, (**b**) SERT, (**c**) MOMA. Data are presented as mean ± SEMs. * *p* < 0.05. Abbreviations: TPH1: tryptophan hydroxylase 1; SERT: serotonin transporter; MAOA: monoamine oxidase A.

**Figure 3:**
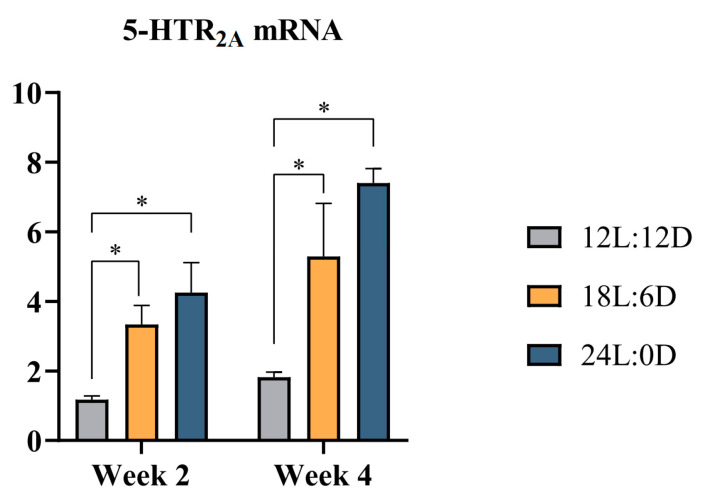
Effects of different photoperiods on 5-HTR_2A_ mRNA expression levels in the breast muscle of broilers. Data are presented as mean ± SEMs. * *p* < 0.05. Abbreviations: 5-HTR_2A_: 5-hydroxytryptamine receptor 2A.

To investigate whether the peripheral 5-HT is affected by different photoperiods, we measured the peripheral 5-HT concentrations in the cecal mucosa, serum, and breast muscle of broilers. As shown in Table 6, compared with the 12L:12D photoperiod, our findings revealed a significant increase in 5-HT concentrations in cecal mucosa, serum, and breast muscle of broilers in the 18L:6D photoperiod and the 24L:0D photoperiod at week 2 and week 4 (*p* < 0.05). Furthermore, to validate the result of higher peripheral 5-HT concentrations induced by long photoperiods, we also performed the mRNA expression levels of 5-HT metabolism-related genes in the cecal mucosa, including TPH1, SERT, and MAOA. As illustrated in Figure 2, compared with the 12L:12D photoperiod, the expression levels of TPH1 and SERT significantly up-regulated in the 18L:6D photoperiod and the 24L:0D photoperiod (*p* < 0.05), whereas there was no effect on the expression level of MAOA at week 2 and week 4 (*p* > 0.05). Furthermore, as demonstrated in Figure 3, compared with the 12L:12D photoperiod, 5-HTR_2A_ mRNA expression levels of broilers in the 18L:6D photoperiod and the 24L:0D photoperiod were significantly up-regulated in breast muscle (*p* < 0.05).

## 4. Discussion

In this study, our findings identified the effects of different photoperiods on peripheral 5-HT, glucose metabolism of breast muscle, and myopathies in broilers. In the present study, exposure to long photoperiods heightened the growth rate of breast muscle and induced the occurrence of WB myopathies. Our observation also indicated that exposure to long photoperiods increased the synthesis and secretion of 5-HT in the cecum, raised peripheral 5-HT concentrations, and up-regulated the 5-HTR_2A_ mRNA expression of breast muscle in broilers. In addition, our findings showed that the accumulation of muscle glycogen decreased, and contents of lactic acid, pyruvate, and L/P increased in the breast muscle of broilers underlying exposure to long photoperiods.

In the current study, we identified the growth-promoting effect of exposure to long-light duration on the breast muscle of broilers. By extending the light photoperiod, the breast muscle weight and breast muscle ratio markedly increased, leading to augmentation of breast muscle growth in broilers. The improvement in breast muscle growth was also observed in previous research on broilers with long day lengths; the breast weights of broilers in the 16L:8D photoperiod were markedly lower than those in the 23L:1D photoperiod [31]. Serval research also showed results that the breast meat yield had a positive correlation or trend with photoperiod [10,32,33,34,35,36]. Moreover, we also revealed that the occurrence of WB myopathies in the breast muscle significantly escalated along with increasing body weight and breast muscle weight of broilers underlying extended light photoperiods. As observed in a previous study, an extended light photoperiod could higher the occurrence of WB in broilers [10]. The rapid growth rate and higher body weight and breast muscle weight of broilers are the most common influencing factors in increasing the occurrence of WB myopathies [5,6]. These findings of the above previous research also support the observation of WS and WB myopathies induced by exposure to long photoperiods in this study.

Here, our findings identified the decreased muscle glycogen, as well as the increased lactic acid, pyruvate, and L/P of breast muscle in broilers. Lactic acid and pyruvate have been reported as the key products and the significant indexes of glycolysis [37]. Although the glucose metabolism in breast muscle under different photoperiods in broilers was still uncertain, a few studies of mice and rats have shown similar results to ours that exposure to different photoperiods strongly modulates the glucose metabolism of skeletal muscle by regulating the glucose metabolism-related gene expression levels [38,39,40]. As one of the markers for mitochondrial function and tissue hypoxia, increased L/P indicates retarded mitochondrial function and exacerbated tissue hypoxia [41], and the increased L/P of breast muscle in the present study was consistent with the results for worsening hypoxia induced by rapid growth [42]. In this study, these findings support the close relationship between extending long photoperiods and glucose metabolism of breast muscle, indicating glycogen accumulation reduction, glycolysis activation, and hypoxic exacerbation in the breast muscle appeared during extended light photoperiods.

The regulation of peripheral 5-HT under different photoperiods is unclear so far. Considering nearly 90% of peripheral 5-HT is produced by enterochromaffin cells in the intestinal mucosa [21,22], each region of the intestinal tract could detect the 5-HT, and the 5-HT concentrations in the cecum were higher in the intestinal tract [19]. This is the first study to identify the potential causal relationship between different photoperiods and cecal mucosa 5-HT metabolism in broilers. TPH1 and MAOA are the rate-limiting enzymes for the synthesis and decomposition of 5-HT in enterochromaffin cells, respectively, and SERT is the most important transporter of 5-HT. By exposure to long light photoperiods, TPH1 and SERT mRNA expression were up-regulated in the cecal mucosa, but there were no effects on MAOA mRNA expression. Our observation clarified synthesis and secretion in cecal mucosa 5-HT metabolism in broilers promoted during an extended photoperiod, while not affecting the decomposition of 5-HT. Moreover, In this study, we also found that peripheral 5-HT concentrations in the cecum, serum, and breast muscle were increased by extending long photoperiods in broilers. These findings could also support the potential causation for exposure to long photoperiods could higher peripheral 5-HT concentrations through up-regulating synthesis and secretion in cecal mucosa 5-HT. In addition, stress could increase the 5-HT concentrations in the intestinal tract [19,20]. Exposure to long photoperiods acts as environmental stress [17,18], which might also induce higher peripheral 5-HT concentrations. However, as there are currently limited experimental studies on the influence of different photoperiods on peripheral 5-HT metabolism, further experimental proof is required in future studies to verify the relationship between photoperiods and peripheral 5-HT metabolism in broilers.

Notably, rapid growth leads to insufficient oxygen supply in meeting muscle growth to induce hypoxic stress and abnormal energy metabolism, which is the main reason for the occurrence of WB myopathies in broilers [5,6,7,8,9]. It has been reported that the occurrence of WB myopathies in broilers appeared simultaneously with abnormal glucose metabolism, including glycogen synthesis reduction and glycolysis inhibition [23,24,43,44,45]. Our observation of lower muscle glycogen, higher lactic acid, pyruvate concentrations L/P, and increased myopathies occurrence provides compelling evidence supporting exposure to long photoperiods induced glucose metabolism disorders, hypoxic exacerbation, and increased WB myopathies.

Intriguingly, we observed an increase in peripheral 5-HT contents, a decrease in muscle glycogen, and higher lactic acid and pyruvate concentrations of breast muscle in broilers under long-day duration, indicating the potential relationship between 5-HT and the physiological responses of breast muscles in broilers during extended photoperiods. Notably, the 5-HTR_2A_ mRNA expression of breast muscle in broilers was up-regulated under extended photoperiods. The role of 5-HTR_2A_ has been reported as an activating receptor for increasing glucose intake and glycolysis in the skeletal muscle cells of rats, influencing the composition of muscle fibers and altering the energy metabolism of skeletal muscles in mice [25,26,27]. This would indicate the potential relationship between 5-HT and the glucose metabolism of breast muscle in broilers during extended photoperiods. However, as the functions of peripheral 5-HT on glucose metabolism were still uncertain, further research is needed to validate these inferences of our research.

## 5. Conclusions

In summary, extending the photoperiod promoted the growth rate of breast muscles in broilers, while reducing muscle glycogen contents, activating glycolysis, and increasing the incidence rates of WB myopathies. It was also confirmed that extending the photoperiod advanced synthesis and secretion of cecal mucosa 5-HT increased the contents of peripheral 5-HT and up-regulated mRNA expression of breast muscle 5-HTR_2A_ in broilers. In this study, a better understanding of the effects of different photoperiods on peripheral 5-HT, breast muscle glucose metabolism, and myopathies in broilers was involved. We are only just beginning to provide a basis for the effects of extended photoperiods on glucose metabolism, myopathies, and peripheral 5-HT metabolism, which could be potential regulatory pathways for improving meat quality in broilers.

## Figures and Tables

**Table 1:** Composition and nutrient levels of basic diets.

Items	5–7 d	8–22 d	23–33 d
Ingredient	Content (%)		
Corn	51.44	54.08	56.85
Soybean meal	40.21	36.82	33.86
Soybean oil	3.94	5.00	5.50
Limestone	1.00	0.85	0.90
CaHPO_4_	1.89	1.80	1.50
NaCl	0.30	0.30	0.30
DL-Methionine	0.21	0. 19	0.19
L-Lysine	0.36	0.32	0.30
L-Threonine	0. 15	0. 14	0.10
Premix ^1^	0.50	0.50	0.50
Total	100	100	100
Nutrient levels ^2^			
ME/(MJ/Kg)	2961	3038	3095
CP (%)	22. 55	21.18	19.97
CF (%)	10.54	10.38	11.35
Ca (%)	0.94	0.85	0.79
AP (%)	0.43	0.41	0.35
Lysine (%)	1.46	1.34	1.25
Methionine (%)	0.54	0.51	0.49
Methionine + cysteine (%)	0.92	0.87	0.84

Abbreviations: ME: metabolizable energy; CP: crude protein; CF: crude fat; AP: available phosphorus. ^1^ Premix provided the following per kg of the diet: 5–7d: vitamin A 12,000 IU, vitamin D3 5000 IU, vitamin E 80 mg, vitamin K3 3.2 mg, vitamin B1 3.2 mg, vitamin B2 8.6 mg, vitamin B6 4.3 mg, vitamin B12 17 μg, pantothenic acid calcium 20 mg, nicotinic acid 65 mg, folic acid 2.2 mg, biotin 0.22 mg, choline 1020 mg, Cu (CuSO_4_·5H_2_O) 16 mg, Fe (FeSO_4_·7H_2_O) 20 mg, Zn (ZnSO_4_·7H_2_O) 110 mg, Mn (MnSO_4_·H_2_O) 120 mg, Se (Na_2_SeO_3_) 0.3 mg, I (KI) 1.25 mg; 8–22d: vitamin A 10,000 IU, vitamin D3 4500 IU, vitamin E 65 mg, vitamin K3 3.0 mg, vitamin B1 2.5 mg, vitamin B2 6.5 mg, vitamin B6 3.2 mg, vitamin B12 17 μg, pantothenic acid calcium 18 mg, nicotinic acid 60 mg, folic acid 1.9 mg, biotin 0.18 mg, choline 1020 mg, Cu (CuSO_4_·5H_2_O) 16 mg, Fe (FeSO_4_·7H_2_O) 20 mg, Zn (ZnSO_4_·7H_2_O) 110 mg, Mn (MnSO_4_·H_2_O) 120 mg, Se (Na_2_SeO_3_) 0.3 mg, I (KI) 1.25 mg; 23–33d: vitamin A 9000 IU, vitamin D3 4000 IU, vitamin E 55 mg, vitamin K3 2.2 mg, vitamin B1 2.2 mg, vitamin B2 5.4 mg, vitamin B6 2.2 mg, vitamin B12 11 μg, pantothenic acid calcium 15 mg, nicotinic acid 45 mg, folic acid 1.6 mg, biotin 0.15 mg, choline 950 mg, Cu (CuSO_4_·5H_2_O) 16 mg, Fe (FeSO4·7H_2_O) 20 mg, Zn (ZnSO_4_·7H_2_O) 110 mg, Mn (MnSO_4_·H_2_O) 120 mg, Se (Na_2_SeO_3_) 0.3 mg, I (KI) 1.25 mg. ^2^ ME determination was performed in the State Key Laboratory of Animal Nutrition and Feeding according to the bionic digestive Operation manual 12L:12DS3, CP content determination was conducted by using a Kjeldahl nitrogen analyzer, and CF content determination was conducted by using a Soxhlet extractor. Other nutrient levels were calculated value according to the Tables of Feed Composition and Nutritive Values in China (2022).

**Table 2:** Primer sequences of target and reference genes.

Gene	Primer Sequence (5′-3′)	Product Length (bp)	Gene Bank Number
GAPDH	F: ACTTTGGCATTGTGGAGGGTR: GGACGCTGGGATGATGTTCT	188	NM_204305.1
5-HTR_2A_	F: TGTCATGCCCGTGTCAATGTR: AGTGGAGAAAAGCACGTCCA	127	NM_001318420.2
TPH1	F: ATGTCTATCGTAAGAGGCGAAAGTAR: TTGGTGAGCAAGGGCAAGTTT	190	NM_204956.2
SERT	F: TCTTCTACTACCTCGTGTCCTCCTTR: GCGGGTATAAAATTCTTCTGCA	155	NM_213572.1
MAOA	F: GCTGCCAAGTTGCTGTATGAGTATR: TCACTTTATAGGTCTCAATGCCCAG	193	NM_001030799.2

Abbreviations: GAPDH: Recombinant Glyceraldehyde-3-Phosphate Dehydrogenase; 5-HTR2A: 5-hydroxytryptamine receptor 2A; TPH1: tryptophan hydroxylase 1; SERT: serotonin transporter; MAOA: monoamine oxidase A.

**Table 3:** Effects of different photoperiods on growth performance of broilers.

Item	12L:12D	18L:6D	24L:0D	SEM	*p* Value
Week 0–2					
Average body weight/g	679.56 ^b^	781.89 ^a^	794.21 ^a^	12.89	<0.05
Breast muscle weight/g	50.35 ^b^	62.81 ^a^	66.43 ^a^	1.89	<0.05
Breast muscle ratio/%	7.31 ^c^	7.89 ^b^	8.51 ^a^	0.15	<0.05
Feed intake per broiler/g	631.11	744.95	911.46	28.34	<0.05
Week 2–4					
Average body weight gain/g	1276.44 ^b^	1414.10 ^a^	1421.39 ^a^	18.48	<0.05
Breast muscle weight gain/g	153.85 ^b^	173.83 ^a^	182.60 ^a^	3.99	<0.05
Breast muscle weight gain ratio/%	12.13	12.43	12.72	0.22	0.576
Feed intake per broiler/g	1589.74	1921.81	2129.4	55.17	<0.05
Week 0–4					
Average body weight/g	1956.00 ^b^	2195.99 ^a^	2215.60 ^a^	27.91	<0.05
Breast muscle mass/g	204.20 ^b^	236.65 ^a^	249.03 ^a^	5.32	<0.05
Breast muscle mass/body mass ratio/%	10.44 ^b^	10.78 ^ab^	11.24 ^a^	0.16	0.109
Feed intake per broiler/g	2188.11	2623.27	2841.93	67.18	<0.05

^a–c^ significantly different within a row with different superscripts (*p* < 0.05). SEM: standard error of the mean.

**Table 4:** Effects of different photoperiods on white striping and wooden breast score of broilers.

Item	12L:12D	18L:6D	24L:0D	SEM	*p* Value
Week 2					
White striping	0.17	0.33	0.17	0.10	0.761
Wooden breast	0.33	0.17	0.50	0.11	0.521
Week 4					
White striping	0.50	0.50	0.33	0.15	0.827
Wooden breast	0.33 ^b^	1.17 ^a^	1.50 ^a^	0.16	<0.05

^a,b^ significantly different within a row with different superscripts (*p* < 0.05). SEM: standard error of the mean.

**Table 5:** Effects of different photoperiods on glucose metabolism in the breast muscle of broilers.

Item	12L:12D	18L:6D	24L:0D	SEM	*p* Value
Week 0–2					
Muscle glycogen/mg/g	5.59 ^a^	4.01 ^b^	2.88 ^c^	0.42	<0.05
Lactic acid/mmol/g	0.75 ^c^	1.02 ^ab^	1.31 ^a^	0.08	<0.05
Pyruvate/μmol/mg	0.19 ^c^	0.22 ^b^	0.27 ^a^	0.01	<0.05
L/P	4.01 ^b^	4.58 ^a^	4.76 ^a^	0.12	<0.05
Week 0–4					
Muscle glycogen/mg/g	5.31 ^c^	3.58 ^b^	2.49 ^a^	0.45	<0.05
Lactic acid/mmol/g	0.86 ^b^	1.38 ^a^	1.55 ^a^	0.11	<0.05
Pyruvate/μmol/mg	0.19 ^b^	0.27 ^a^	0.29 ^a^	0.02	<0.05
L/P	4.56 ^c^	5.13 ^b^	5.38 ^a^	0.14	<0.05

Abbreviations: L/P: lactate-to-pyruvate ratio. ^a–c^ significantly different within a row with different superscripts (*p* < 0.05). SEM: standard error of the mean.

**Table 6:** Effects of different photoperiods on peripheral 5-HT concentrations of broilers.

Item	12L:12D	18L:6D	24L:0D	SEM	*p* Value
Week 0–2					
Cecal mucosa/ng/mg	3.03 ^c^	3.56 ^b^	4.58 ^a^	0.24	<0.05
Serum/ng/mL	19.03 ^b^	23.87 ^a^	26.58 ^a^	1.28	<0.05
Breast muscle/ng/mg	10.02 ^c^	14.19 ^b^	18.62 ^a^	1.31	<0.05
Week 0–4					
Cecal mucosa/ng/mg	2.87 ^c^	4.04 ^b^	4.83 ^a^	0.30	<0.05
Serum/ng/mL	21.35 ^b^	26.38 ^a^	28.92 ^a^	1.31	<0.05
Breast muscle/ng/mg	8.73 ^c^	16.73 ^b^	24.36 ^a^	1.49	<0.05

Abbreviations: 5-HT: 5-hydroxytryptamine. ^a–c^ significantly different within a row with different superscripts (*p* < 0.05). SEM: standard error of the mean.

## Data Availability

The original contributions presented in the study are included in the article, further inquiries can be directed to the corresponding author upon reasonable request.
